# Nitrogen Doped Carbon Nanosheets Encapsulated *in situ* Generated Sulfur Enable High Capacity and Superior Rate Cathode for Li-S Batteries

**DOI:** 10.3389/fchem.2018.00429

**Published:** 2018-09-25

**Authors:** Zhijun Guo, Xiaoyu Feng, Xingxing Li, Xuming Zhang, Xiang Peng, Hao Song, Jijiang Fu, Kang Ding, Xian Huang, Biao Gao

**Affiliations:** ^1^The State Key Laboratory of Refractories and Metallurgy and Institute of Advanced Materials and Nanotechnology, Wuhan University of Science and Technology, Wuhan, China; ^2^School of Materials Science and Engineering, Wuhan Institute of Technology, Wuhan, China

**Keywords:** *in situ* generated sulfur, nitrogen doped carbon, cathode, chemical anchoring, physical trapping, lithium-sulfur batteries

## Abstract

Lithium-sulfur batteries (LSBs), with large specific capacity (1,675 mAh g^−1^), are regarded as the most likely alternative to the traditional Lithium-ion batteries. However, the intrinsical insulation and dramatic volume change of sulfur, as well as serious shuttle effect of polysulfides hinder their practical implementation. Herein, we develop three-dimensional micron flowers assembled by nitrogen doped carbon (NC) nanosheets with sulfur encapsulated (S@NC-NSs) as a promising cathode for Li-S to overcome the forementioned obstacles. The *in situ* generated S layer adheres to the inner surface of the hollow and micro-porous NC shell with fruitful O/N containing groups endowing both efficient physical trapping and chemical anchoring of polysulfides. Meanwhile, such a novel carbon shell helps to bear dramatic volume change and provides a fast way for electron transfer during cycling. Consequently, the S@NC-NSs demonstrate a high capacity (1,238 mAh g^−1^ at 0.2 C; 1.0 C = 1,675 mA g^−1^), superior rate performance with a capacity retention of 57.8% when the current density increases 25 times from 0.2 to 5.0 C, as well as outstanding cycling performance with an ultralow capacity fading of only 0.064% after 200 cycles at a high current density of 5.0 C.

## Introduction

Lithium-sulfur batteries (LSBs) own many advantages including high energy density (2,600 Wh kg^−1^, five times larger than lithium ion bateries), natural abundance, and environmental friendliness (Rehman et al., 2016; Xu et al., 2017; Qu et al., 2018; Zhang et al., 2018). Thus, LSBs have become one of the most promising candidates for large-scale energy storage and long endurance electric vehicles applications (Seh et al., 2016; Chen et al., 2017; Ye et al., 2018). However, the practical implement of the LSBs is impeded by several challenges, such as electronically and ionically insulating of S, large volume change (~80%) during cycling, as well as shuttle effect induced by the high solubility and mobility of the lithium polysulfides (LiPSs) intermediates. To tackle with the above issues, one of the effective routes is encapsulating S into conductive carbon hosts [active carbon (Moreno et al., 2015; Li F. et al., 2017), carbon spheres (Qu et al., 2013; Zhou et al., 2017), graphene (Tang et al., 2016; Du et al., 2017), carbon nanotubes (Li M. et al., 2017) and nanofibers (Liu et al., 2018)] with high porosity or/and high specific surface area to improve electron/ion conductivity, tolerate volumetric expansion and physically trap LiPSs. Nevertheless, the weak interaction between nonpolar carbon and polar LiPSs inevitably results in effusion and irreversible loss of LiPSs from the cathodes and thus rapid capacity fading during cycling. Polar metal oxides, metal sulfides, metal carbides, and metal nitrides have recently been explored as efficient host materials for S cathodes in LSBs and the S/metal oxide composites have been demonstrated to achieve improved cycle stability *via* chemical anchoring between the metal oxide and LiPSs in the cathodes. However, limited active sites of these polar hosts with low surface area lead to low immobilization efficiency for LiPSs (Shi et al., 2018). Recently, heteroatom doping (e.g., N, S, P) has been developed to enable carbon hosts to be polarity and retain the large surface area simultaneously, which can not only trap the LiPSs physically, but also improve conversion kinetics from LiPSs into solid Li_2_S (Wang X et al., 2014; Pang et al., 2015; Zhou et al., 2015a; Wang et al., 2016, 2019; Li C. et al., 2018). Moreover, two-dimensional (2D) nanocarbon materials have drawn special attention as S hosts due to shorter ion/electron transport distance and more active sites (Chabu et al., 2017; Song et al., 2018; Sun et al., 2018; Wang Q et al., 2018).

In this work, we develop a hierarchical three-dimensional (3D) nano-flowers host consisting of hollow 2D nitrogen doped carbon nanosheets (NC-NSs) with micro-porosity shell as the S host. The S is encapsulated in the 2D NC-NSs (S@NC-NSs) with a thickness of 25–40 nm via *in-situ* oxidization of NC coated ZnS NSs (ZnS@NC-NSs) by Fe^3+^. Such unique hierarchical S@NC-NSs assembled by nanosheets endow several advantages as the S host for Li-S: Firstly, the NC shell with typical micro-sized pores can not only physically confine the LiPSs but also chemically anchor LiPS*s via* rich N/O containing functional groups; Secondly, the *in situ* generated S is totally encapsulated in the NC shell, resulting high utilization; Thirdly, S@NC-NSs have large inner space and the elastic carbon shell can accommodate the volume expansion of the S during lithiation; Lastly, 3D hierarchical nano-flowers structure assembled by 2D NC-NSs provides a fast conductive network, and integral structure supporters. Ultimately, the S@NC-NSs deliver a high specific capacity of 1,238 mAh g^−1^ at a current density of 0.2 C (1.0 C = 1,675 mA g^−1^), superior rate performance with a capacity retention of 57.8% when the current density increases 25 times from 0.2 to 5.0 C as well as a remarkable cycling performance with a capacity loss of 0.064% after 200 cycles at a high current density of 5.0 C.

## Experimental

The ZnS NSs were fabricated through a hydrothermal method. Briefly, 0.446 g of Zn(NO_3_)_2_·6H_2_O and 0.114 g of CS(NH_2_)_2_ were added into an aqueous solution containing 20 mL of diethylenetriamine (DETA) and 20 mL of deionized water (DW) under magnetic stirring. Then, the mixed solution was transferred into a 50 mL Teflon-lined autoclave and placed in an oven at 180°C for 12 h. Afterward, the ZnS NSs were obtained by filtration with absolute alcohol and DW for several times. Then, 200 mg of ZnS NSs were dispersed in the 400 mL of DW under magnetic stirring followed by adding 484 mg of Tris-HCl buffer and 400 mg of dopamine (DA) to coat ZnS with polydopamine (PDA) *via* chemical polymerization for 1.0 h under stirring in air. After washing with DW and filtrating for several times, the dried products were further carbonized at 700°C for 2 h to produce ZnS@NC-NSs in N_2_. Then, the ZnS@NC-NSs were dispersed into aqueous ferric chloride (FeCl_3_) solution and stirred over 12 h to convert the ZnS core into S because of the strong oxidizing ability of Fe^3+^. Finally, the reactant was washed with 1 M HCl solution and DW to obtain S@NC-NSs after vacuum drying over night at 60°C.

### Materials characterizations

The morphology, structure and composition of ZnS NSs, ZnS@NC-NSs and S@NC-NSs were characterized by field-emission scanning electron microscopy (SEM, FEI Nova 450 Nano), transmission electron microscopy (TEM, JEM-2100 UHR STEM/EDS), X-ray photoelectron spectroscopy (ESCALAB 250Xi), and X-ray diffraction [XRD, Philips X' Pert Pro (Cu Kα radiation, λ = 1.5418 Å)]. Micromeritics ASAP 2020 analyzer was applied to measure pore size distribution and the N_2_ adsorption-desorption behavior of the NC shell. The S content in the S@NC-NSs was obtained by Naichi Corporation STA449C from room temperature to 600°C with a heating rate of 10°C min^−1^ in Ar. The concentration of LiPSs was determined by Ultraviolet-visible spectrophotometer (CARY 300).

### Electrochemical tests

The S@NC-NSs were mixed with acetylene black and polyvinylidene fluoride (PVDF) with a weight ratio of 7:2:1 to form a homogeneous slurry in N-Methyl-2-pyrrolidinone (NMP). Then, the slurry was uniformly coated on Al foil (15 μm) and vacuum dried at 60°C for 12 h. The coin-type cells were assembled with S@NC-NSs as cathode and Li metal foil as anode. The electrolyte consisted of 1 M lithium bis (trifluoromethane) sulfonimide (LiTFSI) in 1,3 dioxolane/1,2-dimethoxyethane (DOL/DME) (1:1, v/v) containing 0.2 M LiNO_3_. The electrochemical impedance spectroscopy (EIS) in the range of 100 kHz and 10 mHz and the cyclic voltammetry (CV) with a scan rate of 0.1 mV s^−1^ from 1.7 to 2.8 V were conducted on an electrochemical work station (CHI760E). The galvanostatic charging-discharging (GCD) tests were carried out on Neware battery testing system (CT-4008) with different current densities of 0.2, 0.5, 1.0, 1.5, 2.0, and 5.0 C.

## Results and discussions

**Figure 1:**
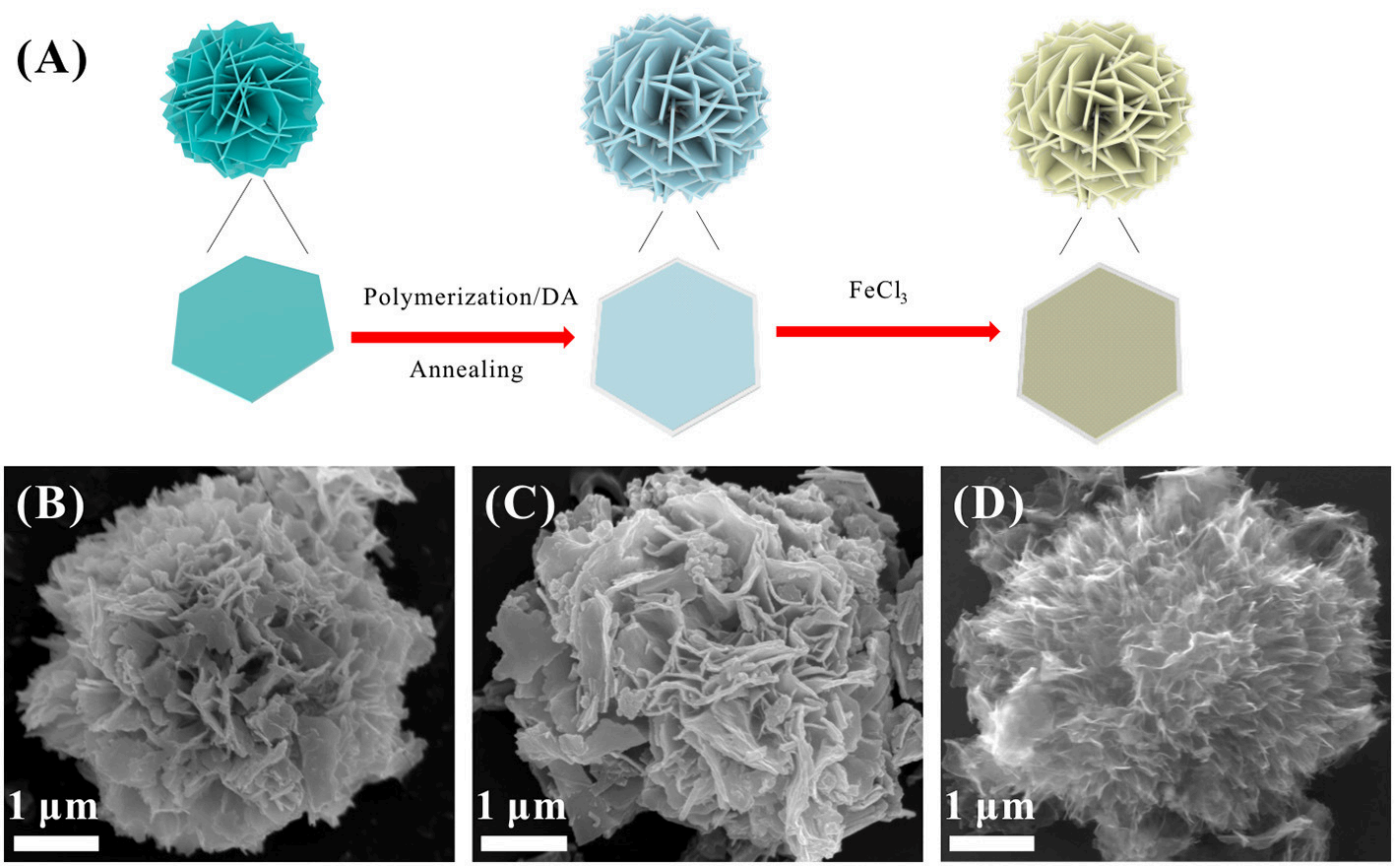
**(A)** Schematic illustration of the synthesis of the S@NC-NSs; SEM images of ZnS NSs **(B)**, ZnS@NC-NSs **(C)**, and S@NC-NSs **(D)**.

**Figure 2:**
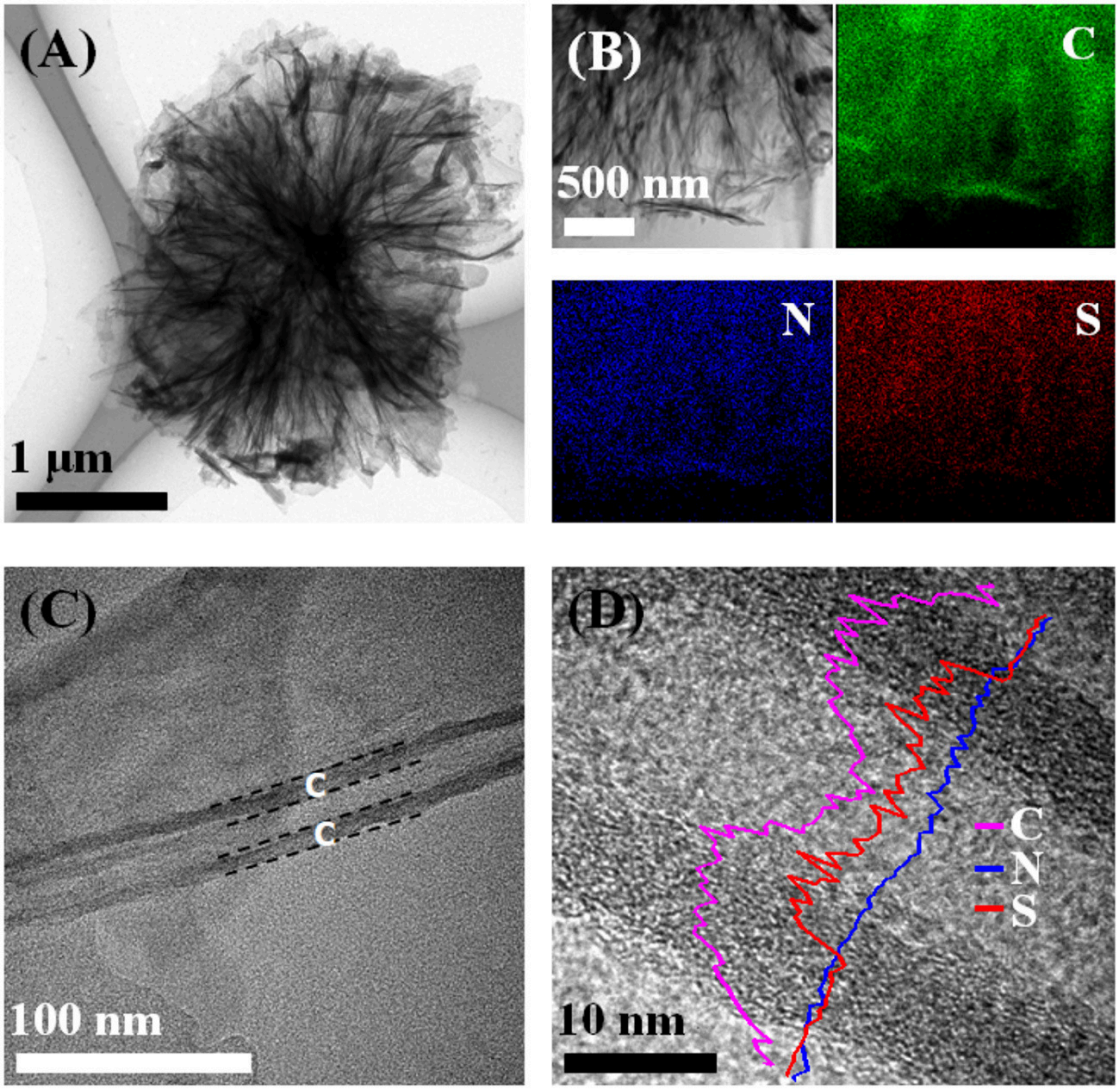
The TEM image **(A)**, elemental mappings **(B)**, HRTEM image **(C)**, and EDS line scan **(D)** of S@NC-NSs.

The synthesis procedure of S@NC-NSs is illustrated in Figure 1A. The ZnS NSs with a diameter of about 3–5 μm (Figure 1B), consisting of 2D nanosheets, were fabricated *via* a hydrothermal route. The XRD pattern of the as-obtained product (Figure S1) can be indexed into the wurtzite phase ZnS (JCPDS Card No. 36-1450). The high resolution TEM (HR-TEM) image in Figure S2A indicates that the thickness of the ZnS NSs is 15–20 nm and discloses a lattice spacing of 0.31 nm corresponding to the (002) plane of wurtzite phase ZnS (Yao et al., 2005). Subsequently, a thin layer of amorphous C is uniformly coated on the surface of the ZnS NSs after the polymerization and annealing strategy. The ZnS@NC-NSs still maintain the flower-like morphology as can be seen from Figure 1C. During the chemical polymerization, PDA can adhere to the surface of the ZnS NSs, and further converted into the NC via annealing in N_2_. The HR-TEM image of the annealed product shows a typical core-shell structure with a crystal core and amorphous shell of 10–15 nm (Figure S2B). All XRD peaks of the carbon coated sample can also be assigned to the wurtzite phase ZnS (Figure S3A). And the XRD pattern (Figure S3B) and Raman spectra (Figure S3C) of the NC shell suggest that the obtained carbon shell is amorphous and has many defects. To further obtain the *in situ* formed S, the ZnS core of the ZnS@NC-NSs was oxidized to S by FeCl_3_ solution according to the reaction of ZnS (s) + 2Fe^3+^ (aq.) = Zn^2+^ (aq.) + S (s) + 2Fe^2+^ (aq.) (Ding et al., 2015; Ma et al., 2017). After oxidization, all diffraction patterns of the sample are corresponded to the orthorhombic S (JCPDS Card No. 08-0247) and no additional ZnS peaks can be observed (Figure S4). Meanwhile, the SEM image in Figure 1D indicates that S@NC-NSs can well preserve the morphology of flowers assembled by nanosheets, which is inherited from that of ZnS and ZnS@NC. Such a microflower structure is further confirmed in Figure 2A. The elemental mappings displayed in Figure 2B indicate that C, N, and S are uniformly distributed in the micro-sized flowers. The TEM image of the S@NC-NSs (Figure 2C) discloses that the nanosheets are hollow double-shell structures with a thickness of 25–40 nm, which provide enough void for the volume expansion of sulfur upon the discharge process. EDS line scan of single S@NC-NSs demonstrates that S mainly exists in the inner wall of the hollow NC, which suggests that the *in situ* formed S is totally encapsulated and adhered to the inner surface of NC shell, as shown in Figure 2D. The mass loading of S in the S@NC-NSs can be optimized by varying the polymerization time. That is, the S@NC-NSs obtained at different polymerization time of 0.5, 1.0, and 2.0 h own different S mass loading of 65, 46.5 and 40.5% (Figure S5A), respectively. Among them, the sample polymerized 1.0 h shows the highest capacity and best cycling stability (Figure S5B), which is characterized in following study.

**Figure 3:**
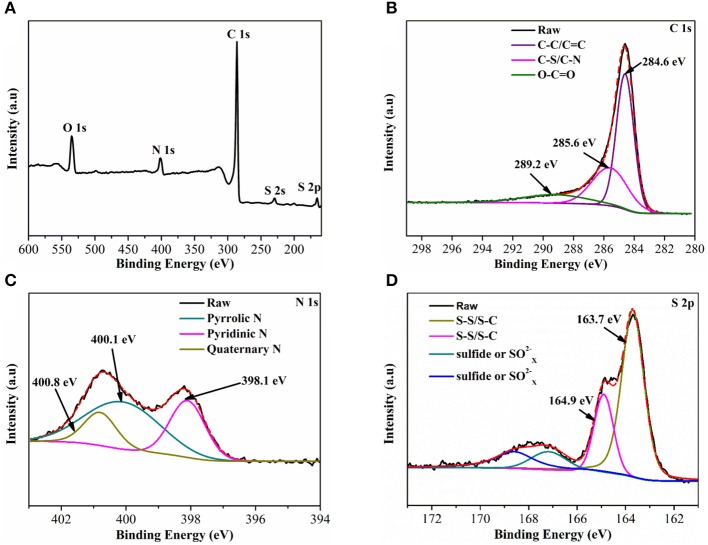
**(A)** Full XPS spectrum of S@NC-NSs; **(B–D)** High-resolution spectrum of C1s, N 1s, and S 2p, respectively.

The X-ray photoelectron spectroscopy (XPS) is used to further analyze the chemical state of the S@NC-NSs. As shown in Figure 3A, the S@NC-NSs contain C, N, O, and S and no observable Zn signal is presented. The C 1s spectrum (Figure 3B) can be devolved into three peaks centered at 284.6, 285.6, and 289.2 eV, corresponding to C–C/C = C (Li et al., 2016), C–S/C–N (Wang Z et al., 2014), and O–C = O (Yang et al., 2016), respectively. It is obvious that there are functional groups containing N/O in NC shell. The N 1s (Figure 3C) could be ascribed to three chemical states: pyridinic N (398.1 eV; Sun et al., 2012), pyrrolic N (400.1 eV; Peng et al., 2016), and quaternary N (400.8 eV; Cai et al., 2017), confirming the effective nitrogen doping of PDA derived carbon. The N doped carbon is beneficial for improving conductivity and wettability (Qiu et al., 2014; Chen et al., 2015). Moreover, in Figure 3D, 163.7 and 164.9 eV are corresponding to S–S/S–C bonds (Zhou et al., 2015b). These functional groups are reported to improve the chemical adsorption ability for LiPSs (See et al., 2014; Song et al., 2014; Chen et al., 2018).

**Figure 4:**
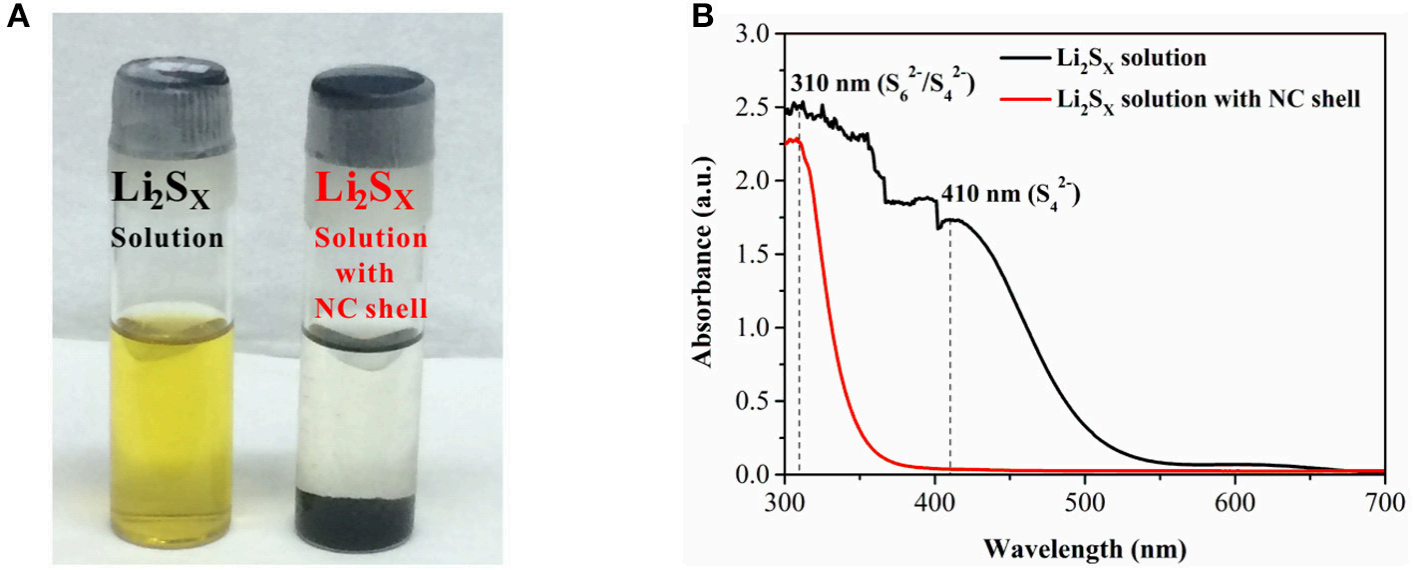
**(A)** Adsorption properties of NC shell in Li_2_S_6_ solution; **(B)** UV–Vis absorption spectra after adding NC shell.

In order to measure the pore structures of the NC shell, the S@NC-NSs were washed in CS_2_ solution to remove the inner S and obtain the pure NC. The pore-sized distribution and the nitrogen adsorption-desorption isotherms of the pure NC are carried out and displayed in Figure S6. The NC shell with a BET surface area of 354.6 m^2^ g^−1^ is mainly consisted of micropores, which is benefit for physically trapping LiPSs. To further evaluate the LiPSs trapping ability of the NC shell, the absorption experiment in orange Li_2_S_6_ solution was conducted. After adding 25 mg of NC shell into 5 mM Li_2_S_6_ solution (4 mL), the orange LiPSs solution turns colorless after 5.0 h (Figure 4A). Moreover, according to the UV–Vis spectra, the characteristic adsorption peaks at 310 (${\text{S}}_{6}^{2-}$/${\text{S}}_{4}^{2-}$) and 410 nm (${\text{S}}_{4}^{2-}$; (Xiao et al., 2015; Li X. et al., 2017)) have been eliminated after adding the NC shell (Figure 4B). Such results imply the strong adsorption capability of NC shell for LiPSs. This excellent adsorption performance is attributed to the physical trapping of the micropores below 2 nm (Figure S6) and chemical immobilization effect of the NC with fruitful O and N containing functional groups (Borchardt et al., 2016; Kang et al., 2016).

**Figure 5:**
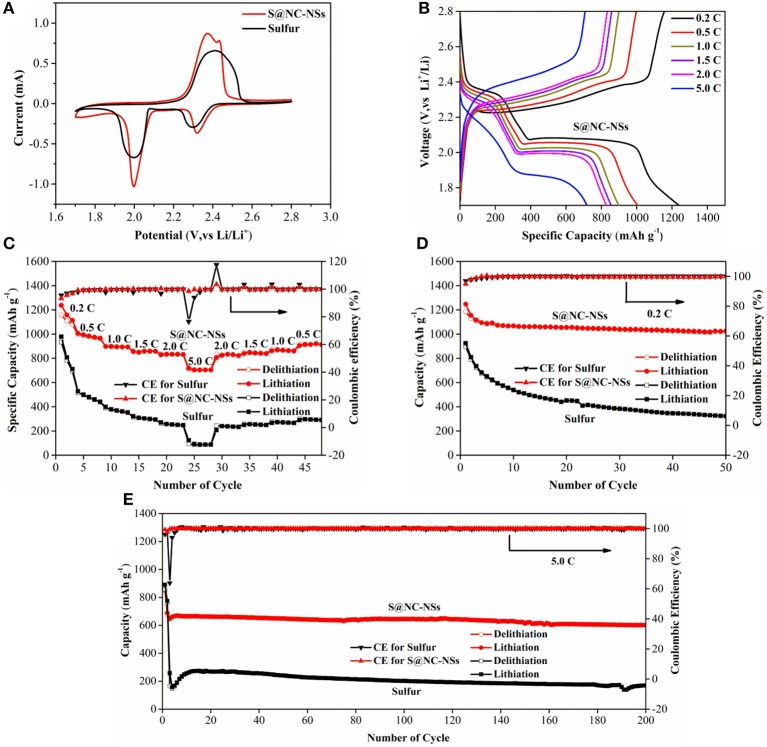
**(A)** CV curves of S@NC-NSs and commercial S; **(B)** Charge/discharge curves of the S@NC-NSs electrode at different current density; **(C)** Rate capability of S@NC-NSs and commercial S; Cycling properties of S@NC-NSs and sulfur electrodes at 0.2 C **(D)** and 5.0 C **(E)**.

To illustrate the remarkable Li storage performance of S@NC-NSs, the coin cell type LSBs were fabricated. And the commercial S was used as the control sample. The cyclic voltammogram (CV) curves of S@NC-NSs and commercial S cathode between 1.7 and 2.8 V at a scan rate of 0.1 mV s^−1^ are presented in Figure 5A. In the cathodic process, the two reduction peaks between 1.98 and 2.30 V can be found in both samples, which are assigned to the formation of soluble long-chain Li_2_S_n_ (4 ≤ *n* ≤ 8) intermediates and solid lithium sulfide (Li_2_S_2_ or Li_2_S). In the subsequent anodic process, the CV curve of S@NC-NSs shows two individual peaks at 2.37 and 2.43 V on virtue of the reactions from lithium sulfide to Li_2_S_n_ (4 ≤ *n* ≤ 8) and S, while the commercial S only shows a broaden peak at 2.42 V because of the large polarization. Compared to the commercial S electrode, it is clearly seen that the oxidation and reduction peaks of the S@NC-NSs electrode are sharper and shift toward the quasi-equilibrium potential, revealing lower polarization and higher reaction kinetics. Figure 5B presents charge-discharge curves of the S@NC-NSs electrode at 0.2, 0.5, 1.0, 1.5, 2.0, and 5.0 C. Two typical plateaus of the S@NC-NSs cathode can be observed at 0.2 C in discharge process delivering a high capacity of 1,238 mAh g^−1^. Even at a high current density of 5.0 C, a remarkable plateau at 1.9 V still exists due to the rapid kinetics of the S@NC-NSs electrode. The capacity retention of the S@NC-NSs cathode is 57.8% when the current density increases 25 times from 0.2 to 5.0 C, which is much better than that of the commercial S electrode, as shown in Figure 5C. The capacity and rate performance of S@NC-NSs are competitive among recently reported S/C composite electrodes (Xia et al., 2017; Zhu et al., 2017; Wang X et al., 2018; Yao et al., 2018). Furthermore, once the rate is reverted back to 0.5 C, the specific capacity of 920 mAh g^−1^ is obtained, revealing its excellent reversibility and rate performance. Figure 5D displays the cyclability of S@NC-NSs and commercial sulfur cathodes at the current density of 0.2 C. Although the S@NC-NSs cathode has an obvious capacity decay at first three cycles, afterwards, it delivers a high and stable capacity of 1,025 mAh g^−1^ over 50 cycles, which is far exceeding that of pure S. Even at a high current density of 5.0 C (Figure 5E), the S@NC-NSs cathode achieves a high capacity of 600 mAh g^−1^ over 200 cycles with a low decay rate of 0.064%. Furthermore, the Nyquist plots of commercial S and S@NC-NSs cathodes after 200 cycles at 5.0 C between 100 kHz and 0.01 Hz are shown in Figure S7. The equivalent impedance of the S@NC-NSs cathode is much smaller than the commercial S cathode, indicating a faster electrons/ions transportation because of special structures of S@NC-NSs assembled by micro-sized flowers with high conductivity. The outstanding cycling stability of S@NC-NSs cathodes can be attributed to dual functions of NC shell with both physical trapping and chemical immobilization of LiPSs.

## Conclusion

In summary, a dual-shell and hollow S@NC-NSs assembled by micro-sized flowers are fabricated for LSBs *via in situ* oxidization process. The *in situ* generated S is encapsulated and adheres to the inner wall of the NC shell with abundant micropores and fruitful N or O containing function groups, which offers both physical trapping and chemical tethering to eliminate the shuttle effect of LiPSs. Moreover, the conjoint hollow NC-NSs also provide high conductive channels for electron transport and enough space for volumetric expansion of S. The S mass loading of S@NC-NSs can be easily adjusted *via* tuning the thickness of the carbon shell. As the Li-S battery cathode, the S@NC-NSs achieve a high capacity of 1,238 mAh g^−1^ at 0.2 C and outstanding rate performance with capacity retention of the 57.8% when the current density increased 25 times from 0.2 to 5.0 C. Importantly, S@NC-NSs demonstrate the excellent stability with a high capacity of 600 mAh g^−1^ and an ultraslow capacity decay rate of 0.064% after 200 cycles at a high current density of 5.0 C. With high conductivity, efficient physical and chemical immobilization as well as adequate inner space, the NC encapsulated *in situ* formed S cathode with outstanding electrochemical performance can bode well for promising application in LSBs.

## Author contributions

ZG carried out the experiment and wrote the paper. BG and XF supervised this research. XL, HS, KD, and XH gave a lot of help for analyzing data. XZ, XP and JF helped writing.

### Conflict of interest statement

The authors declare that the research was conducted in the absence of any commercial or financial relationships that could be construed as a potential conflict of interest.
