# Supplementary material for: Nitrogen Doped Carbon Nanosheets Encapsulated in situ Generated Sulfur Enable High Capacity and Superior Rate Cathode for Li-S Batteries
Source: Front Chem. 2018 Sep 25;6:429. doi: 10.3389/fchem.2018.00429 (PMC6168012; doi:10.3389/fchem.2018.00429)
Supplement: Supplementary file 1 [file Table_1.DOCX]

**Supporting Information**

**Nitrogen Doped Carbon Nanosheets Encapsulated *in situ* Generated Sulfur Enable High Capacity and** **Superior Rate Cathode for Li-S Batteries**

Zhijun Guo ^a^, Xiaoyu Feng ^a^*, Xingxing Li ^a^, Xuming Zhang ^a^, Xiang Peng ^b^, Hao Song ^a^, Jijiang Fu ^a^, Kang Ding ^a^, Xian Huang ^a^, Biao Gao ^a^*

a. The State Key Laboratory of Refractories and Metallurgy and Metallurgy and Institute of Advanced Materials and Nanotechnology, Wuhan University of Science and Technology, Wuhan 430081, China

b. School of Materials Science and Engineering, Wuhan Institute of Technology, Wuhan 430205, China

* Corresponding author:

E-mail: fengxiaoyu@wust.edu.cn (Xiaoyu Feng); gaobiao@wust.edu.cn (Biao Gao)

**Eight pages**

**Figure S1-Figure S7**


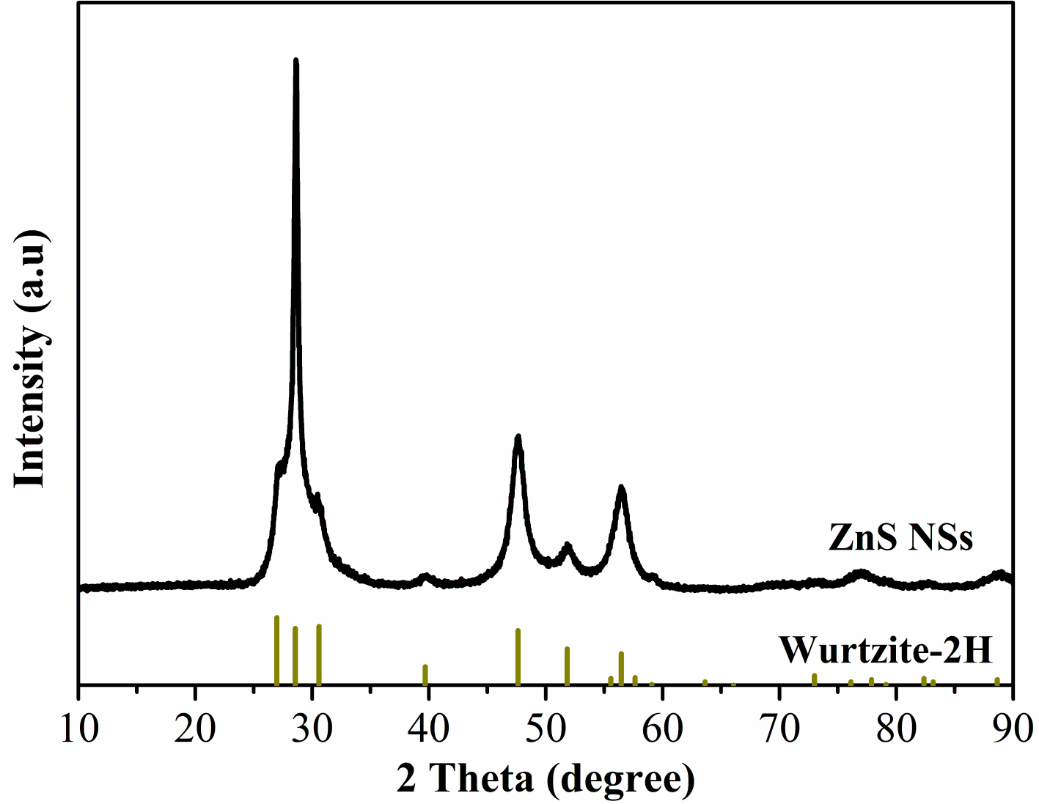


**Figure S1.** The XRD pattern of ZnS NSs.


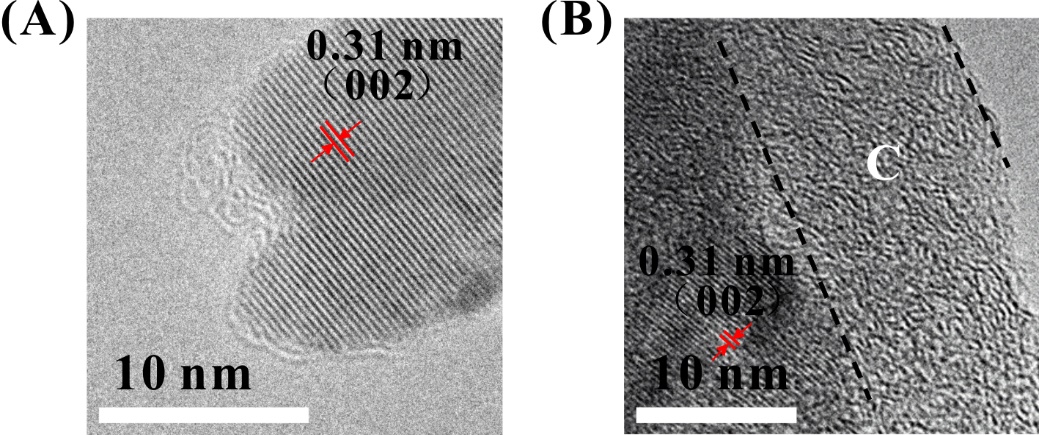


**Figure S2.** HRTEM images of (A) ZnS NSs and (B) ZnS@NC-NSs.


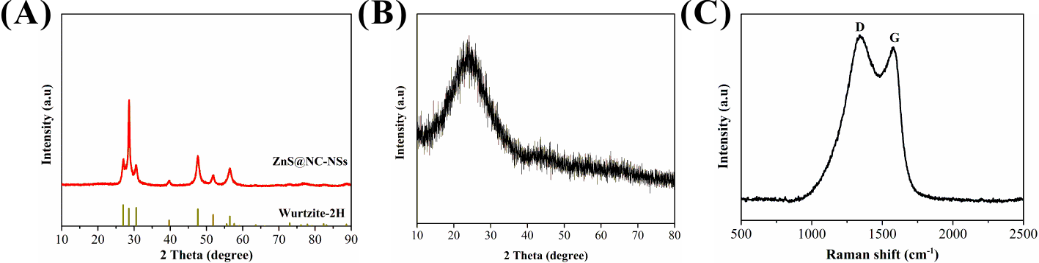


**Figure S3.** (A) The XRD pattern of ZnS@NC-NS; XRD pattern (B) and Raman spectra (C) of NC shell.


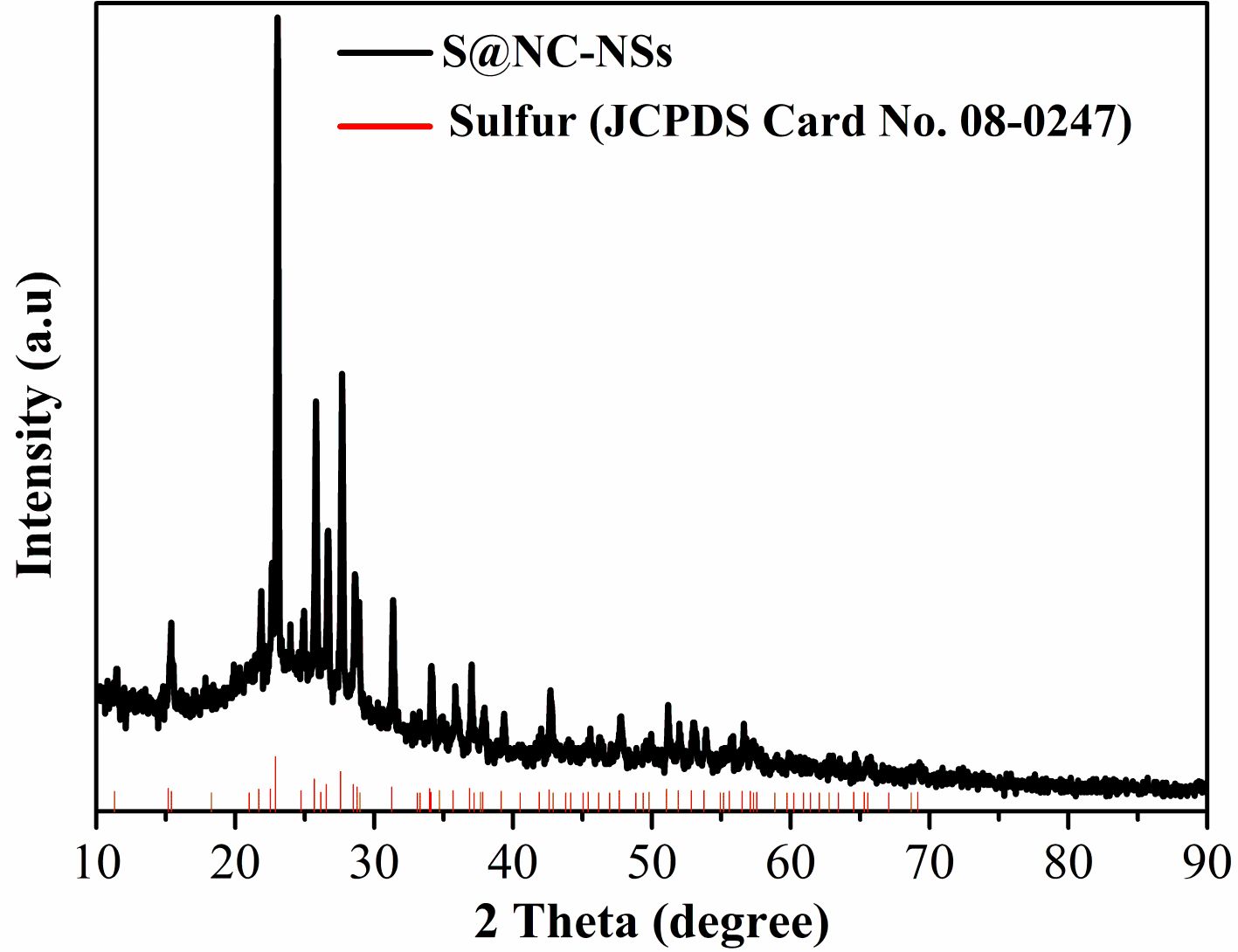


**Figure S4.** The XRD Pattern of S@NC-NSs.


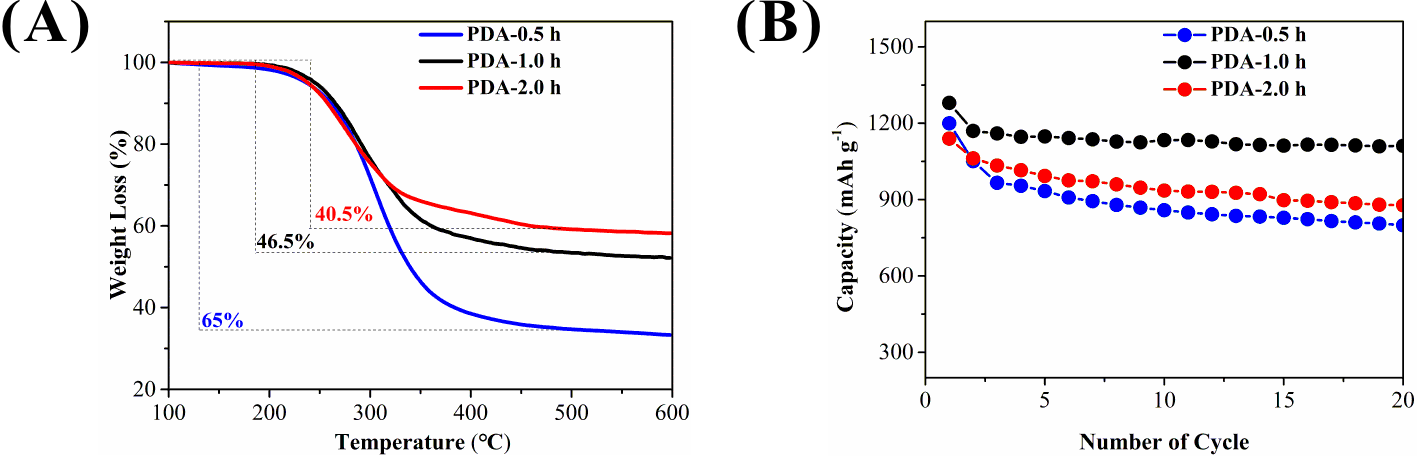


**Figure S5.** (A) TG curves of S@NC-NSs with the different time of dopamine self-polymerization (0.5, 1.0 and 2.0 h), respectively. (B) Cycling performance of S@NC-NSs with different S loading at 0.5 C.


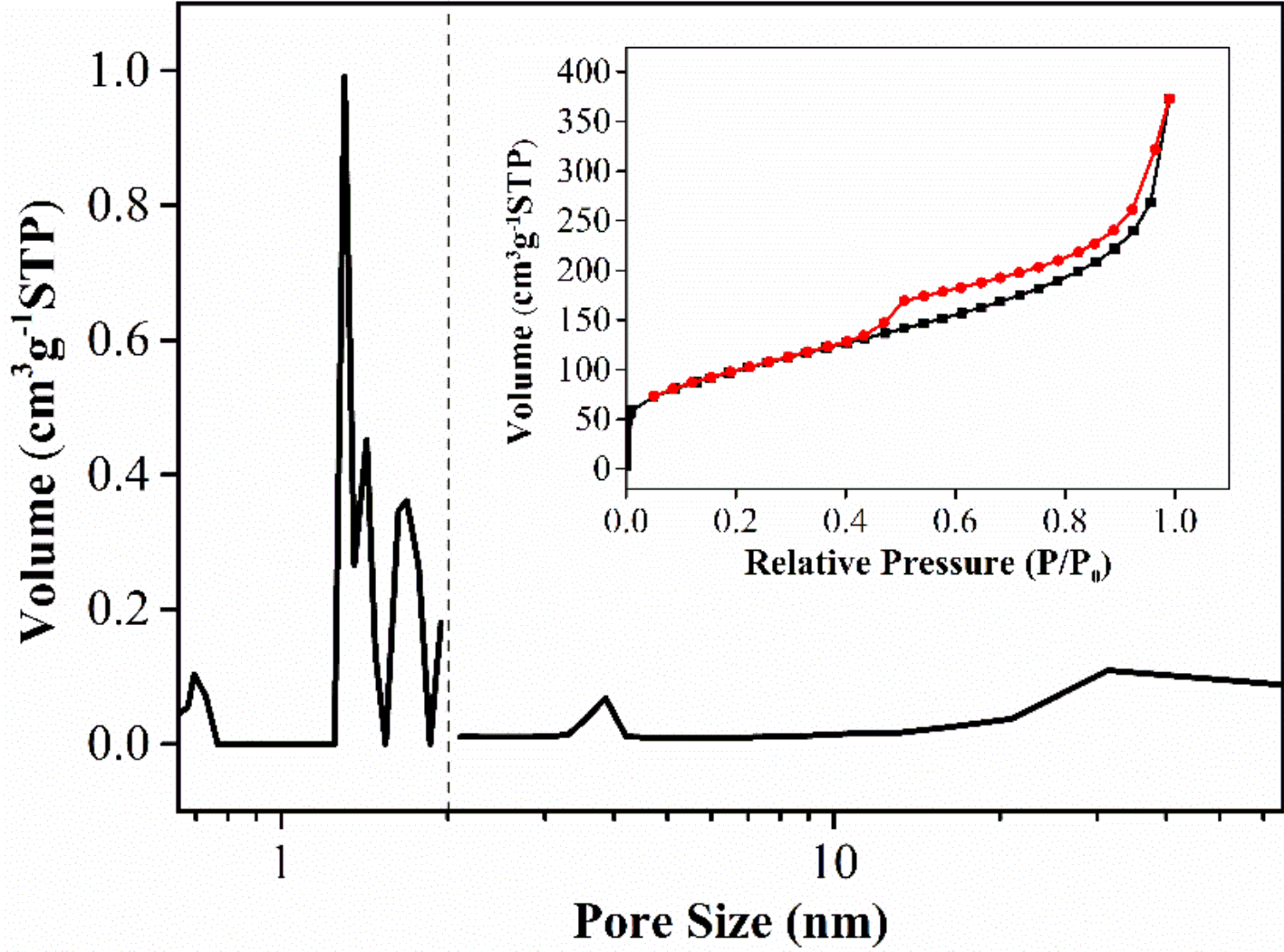


**Figure S6.** The pore size distributions of NC shell with the inset indicating adsorption/desorption isothermals.


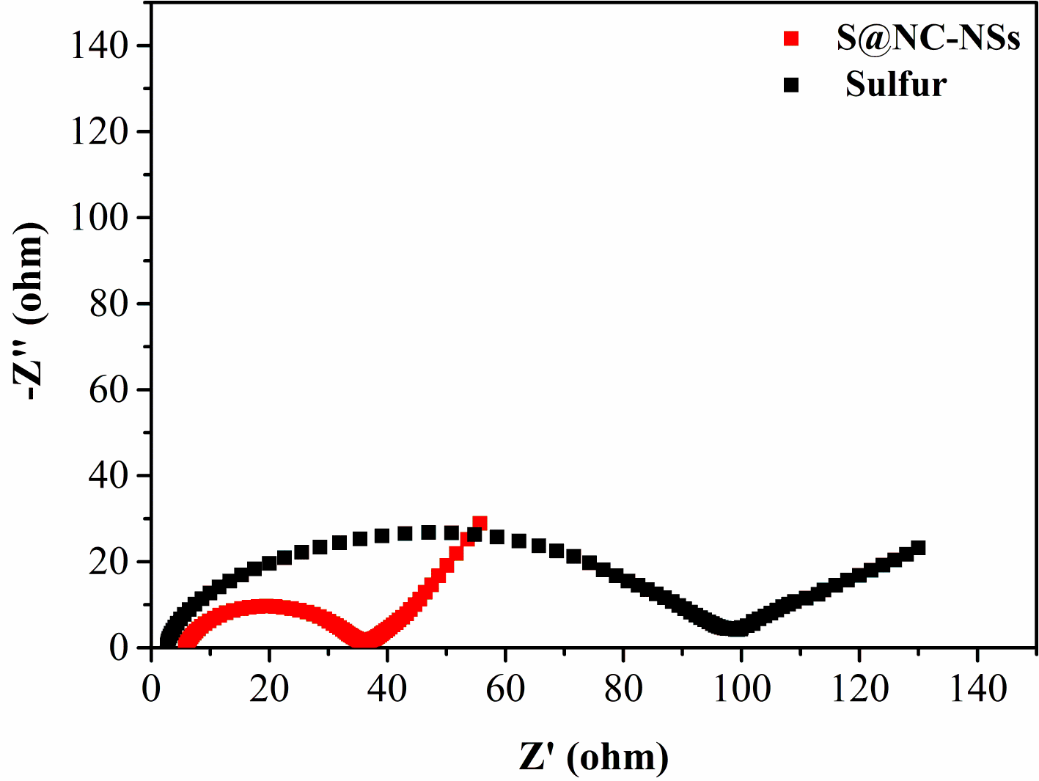


**Figure S7.** The Nyquist plots of commercial S and S@NC-NSs cathode after 200 cycles at 5.0 C between 100 kHz and 0.01 Hz.
